# Primary care nurses’ experiences of how the mass media influence frontline healthcare in the UK

**DOI:** 10.1186/1471-2296-14-178

**Published:** 2013-11-24

**Authors:** Jennifer E van Bekkum, Shona Hilton

**Affiliations:** 1 MRC/CSO Social & Public Health Sciences Unit, University of Glasgow, Glasgow, UK

**Keywords:** Primary care nurses, Healthcare, Mass media, Health visitors, Practice nurses

## Abstract

**Background:**

Mass media plays an important role in communicating about health research and services to patients, and in shaping public perceptions and decisions about health. Healthcare professionals also play an important role in providing patients with credible, evidence-based and up-to-date information on a wide range of health issues. This study aims to explore primary care nurses’ experiences of how mass media influences frontline healthcare.

**Methods:**

In-depth telephone interviews were carried out with 18 primary care nurses (nine health visitors and nine practice nurses) working in the United Kingdom (UK). Interviews were recorded and transcribed. The data was analysed using thematic analysis, with a focus on constant comparative analysis.

**Results:**

Three themes emerged from the data. First, participants reported that their patients were frequently influenced by controversial health stories reported in the media, which affected their perceptions of, and decisions about, care. This, in turn, impinged upon participants’ workloads as they had to spend additional time discussing information and reassuring patients. Second, participants also recalled times in their own careers when media reports had contributed to a decline in their confidence in current healthcare practices and treatments. Third, the participants in this study suggested a real need for additional resources to support and expand their own media literacy skills, which could be shared with patients.

**Conclusion:**

In an ever expanding media landscape with greater reporting on health, nurses working in the primary care setting face increasing pressure to effectively manage media stories that dispute current health policies and practices. These primary care nurses were keen to expand their media literacy skills to develop critical autonomy in relation to all media, and to facilitate more meaningful conversations with their patients about their health concerns and choices.

## Background

With the current emphasis on patient choice in healthcare, mass media such as the Internet, television, print media and radio, plays an important role in communicating and raising awareness about health research and services to patients [1], and in shaping public perceptions and decisions about health [2,3]. It is documented that people obtain most health-related information from the media [4], and that it not only provides information but also sets the agenda for individual and societal discourses [3]. Nowadays social media and more participatory forms of the Internet are playing an increasingly important role in informing and actively engaging patients in healthcare decision-making [5]. In today’s expanding media landscape, people have ready access to a huge array of health information of widely varying quality, complexity and accuracy. How information is reported can influence people’s perceptions of health risks [6] and health-related behaviours [7,8]. During the 2009 swine flu pandemic, the UK news media were found to have provided generally useful and balanced news reports [9], correspondingly public opinion surveys conducted during the height of the summer outbreak found that the public exhibited low levels of worry [10]. In the United States (US) context, the mass media coverage of the hormone replacement therapy clinical trial results, which found increased health risks of cardiovascular disease and breast cancer, demonstrated successful mass media communication [11]. In contrast, media coverage does not always align with the weight of scientific evidence [12] as was demonstrated by UK media coverage of the measles, mumps and rubella (MMR) vaccine scare, which led some parents to lose confidence in the vaccine and to withhold it from their children [7,13].

Indeed, the media have been criticised for presenting information and health risks in an alarmist manner [14,15] and for failing to provide all of the necessary information for people to evaluate risk (e.g. underuse of statistics and few useful comparisons to help people contextualise and understand the personal risk) [14,16]. Whereas historically healthcare professionals were one of patients’ main sources of health and medical information, nowadays people are increasingly involved in accessing online health information for themselves [17,18]. In 2000, an estimated 27.5% of US adults used the Internet to find health-related information [19], a figure which had more than doubled to 61% by 2008 [20]. While the Internet has numerous potential benefits such as empowering patient choice within the decision-making process, there are also concerns about misinterpretation and confusion [21]. A systematic review into Internet health information for consumers found that the quality of information available online was reported as a problem within 70% of the studies [22].

Health professionals themselves also receive health-related information from mass media. For example, nurses in the US reported obtaining information about influenza from media sources such as the TV as well as more official sources such as journals [23]. Within the UK context, Hilton and colleagues [24] found that 77% of health visitors participating in their research reported using the Internet, and 67% the TV, radio and newspapers as sources of information for new research on immunisations. Little is known about how much the media influences healthcare professionals’ behaviours [3]. To date, there are no studies which have explicitly investigated the role that mass media plays in the patient-practitioner encounter from the perspective of primary care nurses working in frontline patient care. This study aims to explore primary care nurses’ experiences of how mass media influences their daily work.

## Methods

### Sampling and recruitment

Between September 2008 and May 2009, eighteen in-depth telephone interviews were conducted with primary care nurses (n = 9 health visitors and n = 9 practice nurses). Using the umbrella term 'primary care nurses’, we invited health visitors and practice nurses as suitable groups of practitioners to take part in the study. Both groups work within primary care settings and as part of multidisciplinary teams, often working autonomously with patients on a one-to-one basis with a broad range of publics and dealing with a wide variety of health issues. Practice nurses’ daily work involves treating small injuries, health screening, family planning, delivering vaccinations and running health promotion interventions. They carry out their work in GP practices and health centres. Health visitors’ daily practice involves offering parenting support on family health and minor illnesses, new birth visits including advice on weaning, feeding and dental health, delivering childhood vaccinations and child health checks. Their work is carried out in patients’ homes and in clinics (http://www.nhscareers.nhs.uk/explore-by-career).

The study’s participants were recruited from across England and Scotland using: posters to advertise the study at the 2007 Community Practitioners and Health Visitors Annual Conference (n = 8), adverts on the Royal College of Nursing website (n = 6), and the technique of snowballing (n = 4). In order to recruit a wide range of practitioners, convenience sampling was used. We aimed to achieve a broad spread in terms of participants’: age; sex, length of experience in the health service; patient caseload characteristics; and geographical location. We also collected information on the number of children that participants had (see Table 1).

**Table 1:** Participant demographics

**ID no.**	**Age**	**Sex**	**Length of service (yrs)**	**Area/caseload**	**Geographical location**	**Number of children**
HV01	60	F	22	Deprived, city	England	3
				High ethnic population		
HV02	32	F	6	Affluent, city	England	None
				High alternative types population		
HV03	53	F	29	Mixed, city	England	3
				High ethnic population		
HV04	49	F	12	Mixed, city	England	2
				High ethnic population		
HV05	49	F	22	Affluent, city	England	2
HV06	63	F	27	Mixed, rural	Scotland	2
HV07	47	F	16	Mixed, rural	England	2
				High alternative types population		
HV08	61	F	36	Mixed, rural	England	2
HV09	52	F	26	Mixed, rural	Scotland	1
PN01	39	F	6	Deprived, rural	England	2
PN02	34	F	8	Mixed, rural	England	5
PN03	44	F	19	Mixed, city	England	None
PN04	36	F	2.5	Mixed, city	England	1
PN05	28	F	7	Affluent, city	England	None
PN06	55	F	30	Deprived, rural	Scotland	None
PN 07	50	F	8.5	Mixed, rural	Scotland	3
PN08	59	F	20	Mixed, city	England	3
PN09	49	F	17	Affluent, city	England	4

### Instrument

The semi-structured interview was chosen as an appropriate data collection method, allowing for flexibility in the interview guide and enabling the researchers to develop an in-depth understanding of the research topic [25]. The interview guide covered five broad topic areas: demographic details (e.g. patient caseloads and experience); sources of information that participants currently use; conflicting evidence; confidence; and assessing research evidence. Probes were used to encourage participants to speak in more detail about relevant topics.

### Data collection

The study was granted ethical approval from the NHS National Research Ethics Committee. Before each interview, informed consent was obtained from participants. All interviews were conducted over the telephone and lasted between 42 and 81 minutes, with an average time of 58 minutes. Using telephone interviews was a convenient technique for gathering data as the participants had very busy workloads and were spread across different locations. We found that the use of telephone interviews yielded rich data as practitioners were able to speak openly about the challenges they faced [26]. Four participants carried out their telephone interviews from their workplace and the remaining 14 participants from their own homes. One disadvantage of conducting interviews over the telephone is the absence of non-verbal cues that can make this method more difficult. However, the researcher carrying out the interviews was experienced in this technique.

### Data analysis

All of the interviews were audio recorded and then transcribed. Participants’ anonymity was ensured by replacing each person’s name with an individual code throughout the transcripts. Tentative themes were developed through the iterative process of reading and re-reading each transcript [27]. Next, the data was imported into NVivo 9 software, and the principles of constant comparative analysis within and between transcripts were used to further develop and refine the themes [28]. To enhance the credibility and the quality of the themes, two of the study’s researchers worked together on a 'depth perception’ exercise [29]. This facilitated a more critical analysis of the participants’ accounts by asking 'why’ questions about the content, and discussing theoretical links to the data. As a result of this exercise some small changes were made to the themes.

## Results

Three themes emerged from the data. These were: 'mass media influence on patients’ , 'mass media influence on nurses’ and 'developing media literacy skills’.

### Mass media influence on patients

During the interviews, it was common for nurse participants to speak about their patients being heavily influenced by the Internet, newspapers and television reporting on health topics. It was common for participants to suggest that mass media reporting can cause patients to develop anxieties about particular health issues and risks, or to draw attention to health-related scientific breakthroughs that may not be evidenced or recommended by the Department of Health. As a result, participants mentioned that frequently patients would come to them seeking further explanations or reassurance about particular topical health issues. One health visitor stated: “when there’s something been on the news you knew you’d get phone calls the next day about that thing” (HV05). Participants spoke of the challenges of dealing with patients that have been exposed to inaccurate or conflicting media reports that deviate from current practice or advice. Looking back, the MMR vaccine scare was noted as a good example of this. For instance, one nurse said:

The evidence that was being presented in the media wasn’t the, you know the- what was correct. So that was probably it but it’s still quite difficult to explain that to people and I think people have the perception that you’re, you know, you just want them to have the vaccine because, you know, that’s what you’re getting paid to do (HV07).

Several participants expressed frustration about their patients’ exposure to health stories via mass media because of the consequences on their workload. For instance one practice nurse stated:

“…some people, they do just book appointments now just to discuss, you know, what they read in their Sunday paper and you just think, 'Well really, you know, sort of everyone’s busy enough as it is, you know, appointment-wise without having to do that” (PN05).

Across the interviews there was some discussion about the differing forms of mass media consumption by different patient groups. Generally, participants thought that patients from higher socio-economic groups consumed health information from the Internet. They were more likely to be educated, and well-informed about the differing topical debates, which could result in time consuming conversations that challenged current practices and advice. In contrast, patients from lower socio-economic groups, described as less educated, were perceived as more likely to read articles in tabloid newspapers as a source of health information. However, they were equally deemed to impact on participants’ time and workload because of their increased exposure to poor quality news reporting and their lack of confidence in judging how to make sense of these news reports. As one practice nurse commented:

…I think young mums, you know, see the headlines, I think the media doesn’t help, the papers I feel and the television, the media tends to blow it up even more, I feel that. They aim to sell papers, isn’t it, and to, you know, get viewings on the news. I think it’s slightly cruel because it puts that doubt in a parent’s mind (PN08).

### Mass media influence on nurses

Many participants discussed times during their careers when news media stories evoked personal anxieties about healthcare practices or treatments, and spoke of finding it difficult when media stories reported contradictory evidence to current practice. Participants often referred to the media coverage surrounding the MMR vaccine debate and how this undermined their own confidence in the safety of the MMR vaccine. One health visitor summed up the general view: “It was really difficult because there was so much conflicting information out there, and at the back of my mind I had this nagging doubt that, there could have been something inherently wrong with the MMR vaccine” (HV03).

It was commonly recognised that mass media stories can present biases, inaccuracies and opinion-based views. However, several of the participants contrasted their maternal and protective instincts evoked by emotive news stories about children with their need to try and remain objective about approved practices.

I mean if you’re reading a newspaper and you see an article about this poor child that was damaged, it’s gonna grab your attention and it’s gonna tug on your emotions, it’s gonna bring [out] the parent in you… But then if I saw another article that was loads of facts and figures, you don’t have that emotional connection with it do you? (PN02).

Many participants alluded to a dissonance between their intuitive and rational feelings, especially when there were conflicting media reports about evidence and ethical issues such as patient safety. Participants often talked about looking back over their careers recalling experiences when there was a disconnect between the recommended practice and mass media reports raising concerns about those practices. For example, one health visitor stated:

… next time, sort of say I’m going to be much quicker to sort of back [the] Department of Health’s stance, and the stance that I need to take as a health professional… I think I’ve become more savvy of the media as a result (HV03).

Participants were aware of some of the pitfalls of mass media stories and reported possessing varying degrees of appraisal skills. However, due to working in a time-pressured environment they commonly spoke of relying on daily news in newspapers, on the television and on the Internet to inform them about new research and health discoveries, and to keep abreast of new developments in healthcare. Many practitioners did discuss reading academic journals, but few stated that they used them to keep up-to-date with new research. One reason offered was that some found journal formats difficult to make sense of and decipher practical implications from. While all the participants mentioned that they did receive information through official channels such as the Department of Health, a common criticism was that this information often came in response to the media and after they had had to deal with concerned patients. For example one health visitor stated:

Well, we’re either one step behind the parents or we’re at the same level as the parents because if they hear about it in the Press, so do we… we do get information down from the Department of Health and we do have information coming on updates, but what we find is it often comes too late, you know, because things come out in the media before we get our information down through the professional channels (HV08).

Only two participants spoke of their Healthcare Trusts being effective at providing new information in a timely manner. One practice nurse explained why this was the case in her practice: “Our practice manager is very good. Anything that’s relevant to us that’s sent to her by email or through the post, she will automatically always pass it on to us”(PN01). However, she spoke of how this was not the official line of communication at her workplace and that the Primary Care Trusts or the Department of Health should be responsible for sending out this type of information to healthcare professionals.

### Developing media literacy skills

Some participants talked about the need for a more critical understanding of how mass media works in order to help them make sense and communicate with patients about media reports. One practice nurse stated:

…creating something for health professionals [so] that they are able to become a little bit more savvy to the way that journalists work might help them when the next sort of health controversies happen. Just to understand what it is, or where it’s come from and actually what the reality is rather than what the headlines say (PN07).

It was suggested that such educational materials would need to explain in simple language how to interpret the information presented in Internet, print media and television reports. For instance, one practice nurse said:

I think there’s a growing issue now that maybe 20 years ago, you know, there’s been a sort of change in the way that, you know, that health sells newspapers and people are interested in health, and health professionals need help to deal better with those kind of, just having an understanding, even maybe of the way that journalists work and stories get broken [down] and all those kind of a things. I just think you know if even we just had the little leaflet just to give the patient which just explained, you know, just a general thing to give them an idea that, you know, just because they’ve read it, doesn’t mean it’s true (PN05).

Some participants expressed concern about their relative lack of confidence in judging how to make sense of, and communicate about, new discoveries or conflicting news reports. It was found that there is a current desire for a resource to help participants and their patients understand issues around biased or misleading mass media reporting, especially when conflicting with current recommended practice:

I think we could have something developed that is quite simple that gives you more-maybe sort of help to sort of see, to critically appraise the media rather than necessarily from–geared towards critically appraising research (PN01).

Only one participant reported having had any training in communicating about health issues and media reporting (HV03), and over half of the participants thought that some form of media training or the development of a resource would be beneficial to their professional practice.

## Discussion

In line with previous research, the primary care nurses who participated in this study reported that patients could be heavily influenced by controversial health stories from various forms of mass media such as the Internet, print media and television [3,30,31]. This not only impacted on patients’ judgments about some healthcare practices, but also impinged on primary care nurses’ workloads as they reported having to spend an increasing amount of time discussing the latest news stories with patients, especially when controversies occurred. With the increasing availability of unregulated information and the drive for open-access journal articles online, a wide spectrum of health literature, which varies in accuracy, complexity and quality, is available to the public. This, coupled with the patient choice agenda in healthcare, empowers individuals to form decisions themselves [32,33]. An emerging issue, then, that needs to be addressed is: should healthcare professionals embrace the increasing task of engaging and aiding patients with the appraisal of self-sourced health information? Research suggests that people who use the Internet to source health information need more help from healthcare professionals with interpreting and understanding reports to be able to make choices themselves [34,35]. Nurses are centrally positioned to help advance patients’ knowledge and decision-making about health information, and, in turn, to help improve their health outcomes [36,37]. However, in an age where people experience an overload of information from mass media channels, providing this support to patients appears to be taking its toll on primary care nurses’ workloads. If healthcare professionals are to effectively support patients in deciphering media stories, they not only need to be up-to-date with accurate information and evidence-based practices, but they also need to feel confident in their own abilities to appraise and contextualise mass media reports. Of some concern is the finding that media reports were often cited as primary care nurses’ first point of contact with new or controversial health information due to time pressure, easy access and a lag in dissemination from official channels. Previous research has also found that nurses use mass media as a source of evidence [24]. In view of this, healthcare professionals should be encouraged and given time to access and engage with original research (which media stories are often based on) so that they are able to appraise the primary source of information. While critically appraising evidence is an important aspiration for healthcare professionals, research suggests nurses are often unable to find the time, and sometimes lack the skills, to do so [24,38-40]. Therefore, we encourage official channels such as the Department of Health and Primary Care Trusts along with academic researchers to work towards more rapid and effective dissemination and engagement of evidence summaries and statements to staff working at the frontline of healthcare services. These summaries and statements can be easily consumed by both healthcare professionals and their patients. It has been suggested that social media such as Twitter and online forums, which are being increasingly used by official agencies, can prove essential aspects of communication strategies if used effectively [41]. Social media can also provide valuable opportunities for intra-professional communication within healthcare [42], which will enable nurses to gain accurate, real-time updates. We also encourage healthcare professionals to make use of existing official resources, such as the 'Behind the Headlines’ section of the NHS Inform website, which provides unbiased up-to-date quality-assured health information (http://www.nhsinform.co.uk/behind-the-headlines.aspx).

Tabloid newspapers, found to produce lower quality, less-informed health stories in greater frequency than broadsheet newspapers [43], were reported as a common source of health information for lower socio-economic patient groups. Alarmist news stories that present public health risks can have a negative impact on audience’s behaviour [15]. Our findings suggested that nurses believed these groups of patients may need more support in making judgments and decisions about care. Alternatively, patients from high socio-economic groups, who were viewed by these nurses as educated, and who appeared to have researched and deliberated on a number of online information sources, would frequently challenge and decide not to follow recommended practice. One explanation proposed is that people’s socio-cultural group identities can also lead to biases in decision-making, as individuals align their views with specific media messages that are congruent with their identities but that do not necessarily support the objective evidence [44]. Therefore, being more educated does not imply that decision-making will result in the most informed conclusion. To the contrary, researchers in the US [45] found that people who are more likely to spend time deliberating on their decisions about the health risks of the human papilloma virus vaccine (a health topic that became highly politicised in a number of States) did not all reach the most informed conclusion. Instead, their views became more polarised to opposite extremes, depending on their cultural identities and political persuasions, as opposed to aligning with the scientific evidence.

Mass media can play a role in the social amplification of health risks, too [46,47], whereby experts assess practices or treatments as relatively low risk but they take on social and political identities fuelled by the media, which amplifies their risk disproportionately [48]. In our study, primary care nurses often referred to the MMR controversy played out in the media as having a significant negative impact on vaccination uptake; the repercussions of which are being felt a decade later with recent measles outbreaks in parts of the UK.

It is important to note that while the media can influence the public in the formation of social-level judgments, studies have shown that people often rely on interpersonal channels such as social networks, family and friends to help shape their perceptions of health risks [49,50]. However, some research indicates that, especially during high levels of publicity, health stories in the media can be more influential than interpersonal sources [30]. This indicates that providing accurate and up-to-date information to counterbalance inaccuracies in media stories may assist health professionals to confidently discuss and share best evidence with patients, while taking account of their personal views and preferences.

In our findings, primary care nurses were aware of the common pitfalls of mass media reporting, but sympathised with patients about the alarmist and fear-evoking nature of some news reports. They too recalled times in their own careers when media reports contributed to a loss in their confidence and trust towards certain healthcare practices and treatments. The finding that the media can directly influence primary care nurses’ own perceptions of health risks has scarcely been reported. Although healthcare professionals are expected to use critical appraisal skills to interpret health information, it has been acknowledged that emotions can override analytical reasoning [51]. All of the participants who took part in our study were female, with over three quarters being mothers, and some discussed being torn by strong maternal and emotional instincts when confronted with an influx of media stories reporting on unsafe treatments for children. Emotional stories were discussed as being more powerful and engaging than dry facts and figures. In the psychological literature, emotion is widely considered to play a core role in decision-making, as people form judgments not only from what they think but also from what they feel [52]. Dual processing theory [53] proposes that there are two systems at work in the formation of judgments and decisions. The intuitive system is fast-acting, automatic, emotion- and intuition-based, heuristic-forming, experiential and unconscious, while the deliberative system is slower-acting, cognitive, rational, logical, analytical and conscious. In evidence-based medicine, there is a strong reliance on rational, critical and scientific inquiry, which aligns with deliberative thinking and with patients being encouraged to use deliberative and analytical processes to appraise options [54]. Within the wider literature, the importance of using intuitive and experience-based tacit forms of knowledge in decision-making is recognised [55-58], with evidence of people using both intuitive and deliberative thinking to arrive at a decision [59]. Although there may be some valid arguments for incorporating intuitive thinking into nursing practice, there are risks associated with it and this should not be at the expense of delivering safe and effective healthcare and advice [57].

Our findings indicate that in an age that is characterised by a growing availability of information, primary care nurses felt they had little support to expand their own media literacy skills and to engage and develop these skills in their patients. While media literacy is still a relatively new field of inquiry, a recent review on its effectiveness reported positive outcome effects on: media knowledge; criticism; perceived realism; influence; behavioural beliefs; attitudes; self-efficacy; and behaviour [60]. Media literacy training would provide an overarching and more critical understanding of the way in which media messages are produced and framed. Providing more educational training and resources, aimed at developing deliberative thinking, will not provide a 'magic bullet’ solution to eradicating the negative influences of media reporting, as judgement and decision-making is also affected by other personal, socio-cultural, and political factors (discussed above). However, the fundamental goal of media literacy is to maintain critical autonomy in relation to all media [61]. Therefore, providing individuals with the critical and analytical tools to better decipher media messages will help to: ameliorate uninformed decision-making; empower to promote better self-management of patients; instill more confidence in health professionals to trust best evidence guidelines during health controversies; and facilitate more meaningful and effective conversations between patients and healthcare professionals about their health concerns and choices. On a final note, although this paper primarily focused on the potential negative influences of mass media on patients and health professionals, it is important to recognise that responsible, well-informed media reporting can be an asset [9,11,62]. Effective mass media communication can deliver important messages, facilitate public engagement in health sciences, support better decision-making in health matters and help to save lives [63].

### Strengths and limitations

So far, little research has been carried out on primary care nurses’ experiences of how mass media can affect their daily practices and perceptions about the healthcare that they provide to their patients. This study used qualitative interviews to provide descriptive, detailed data on the subjective experiences and views of primary care nurses. Qualitative methods are recommended when a topic is relatively unexplored, as was the case in our study, and can provide contextually bound in-depth accounts. However, it is important to recognise that qualitative methods are limited in their generalisability. Another potential limitation of this research is that the health visitors and practice nurses who took part in this study were self-selected and may represent a highly engaged group within their professions. There are also some potential differences between the roles and settings of health visitors and practice nurses, as health visitors would primarily carry out house visits and focus on child health and practice nurses would usually be situated in doctors’ practices or health centres working with a wider cross-section of the public. These contextual differences could alter the relationships that health visitors and practice nurses have with their patients but in relation to media influences this did not seem to be the case from analysing their accounts. It is also important to note that the age of the data (collected in 2008/2009) is likely to present a somewhat dated picture of how more contemporary forms of media, such as Facebook, Twitter, blogs etc. operate within the healthcare environment today.

## Conclusion

In conclusion, the findings from this paper indicate that healthcare professionals can be negatively influenced by mass media stories, especially at times when health controversies occur. In an ever expanding media landscape and an era of patient-choice, the potential for the influence of the media to negatively impact patients’ decisions and the quality of healthcare they receive needs to be taken seriously. National health organisations and academic researchers need to ensure that they are rapidly disseminating quality-assured information and effectively engaging with healthcare professionals and the public. One way of doing this could be to make more use of social media platforms. Additionally, healthcare professionals should be encouraged to use existing official information resources aimed at making sense of media reports. We acknowledge that providing accurate information alone will not necessarily prevent patients from making biased judgements, due to the many other factors involved in decision-making. Nevertheless, it is important that healthcare professionals feel confident to discuss media reports and best practices with patients, while taking account of patients’ personal views and preferences. Healthcare professionals’ own intuitive and emotional responses can form part of their professional practice, but they should ensure that using tacit knowledge is not at the expense of delivering safe and effective healthcare and advice. Nurses working in primary care face increasing pressure to effectively manage media stories that dispute current health policy and practice. These primary care nurses were keen to expand their media literacy skills to develop critical autonomy in relation to all media, and to facilitate more meaningful conversations with their patients about health concerns and choices.

## Competing interests

We have no competing interests. This study was funded by the MRC/CSO, Population Health Science Research Network and conducted in the Understandings and Uses of Public Health Research programme (25605200 68096) at the MRC/CSO Social and Public Health Sciences Unit, University of Glasgow. The funding body had no role in the design, collection, analysis or interpretation of this study.

## Authors’ contributions

JvB participated in the analysis and drafting of the manuscript. SH participated in the design, data collection, analysis, and in drafting the manuscript. Both authors approved the final manuscript.

## Pre-publication history

The pre-publication history for this paper can be accessed here:

http://www.biomedcentral.com/1471-2296/14/178/prepub

## References

[B1] Atkin C Wallack L Mass communication and public health 1990 Newbury Park: Sage Publications

[B2] Liu H Priest S Understanding public support for stem cell research: media communication, interpersonal communication and trust in key actors Public Underst Sci 2009 18 704 718 10.1177/0963662508097625

[B3] Grilli R Ramsay C Minozzi S Mass media interventions: effects on health services utilisation Cochrane Database Syst Rev 2002 CD000389 PMID: 10796539 11869574 10.1002/14651858.CD000389 PMC13429285

[B4] Schwitzer G Ten troublesome trends in TV health news Br Med J 2004 329 7478 1352 10.1136/bmj.329.7478.1352

[B5] Jones S Fox S Generations online in 2009 2009 Washington, DC: Pew Internet & American Life Project

[B6] Kitzinger J The media and public risk 2009 London: The Risk and Regulation Advisory Council

[B7] Hilton S Petticrew M Hunt K Parents’ champions vs. vested interests: who do parents believe about MMR? A qualitative study BMC Public Health 2007 7 1 42 10.1186/1471-2458-7-42 17391507 PMC1851707

[B8] Hilton S Hunt K Bedford H Petticrew M School nurses’ experiences of delivering the UK HPV vaccination programme in its first year BMC Infect Dis 2011 11 1 226 10.1186/1471-2334-11-226 21864404 PMC3176210

[B9] Hilton S Hunt K UK newspapers’ representations of the 2009-2010 outbreak of swine flu: one health scare not over-hyped by the media? J Epidemiol Community Health 2011 65 941 946 10.1136/jech.2010.119875 21131303 PMC3171979

[B10] Rubin G Amlot R Page L Wessely S Public perceptions, anxiety and behaviour change in relation to the swine flu outbreak: cross sectional telephone survey Br Med J 2009 339 b2651 10.1136/bmj.b2651 19574308 PMC2714687

[B11] Canales MK Breslau ES Nelson DE Ballard-Barbash RR Did news reporters get it right? Translation of the 2002 hormone study findings Am J Prev Med 2008 34 1 61 68 10.1016/j.amepre.2007.09.023 18083452

[B12] McComas KA Defining moments in risk communication research: 1996-2005 J Health Commun 2006 11 1 75 91 10.1080/10810730500461091 16546920

[B13] Begg N Ramsay M White J Media dents confidence in MMR vaccine Br Med J 1998 316 561 10.1136/bmj.316.7130.561

[B14] Rowe G Frewer L Sjöberg L Newspaper reporting of hazards in the UK and Sweden Public Underst Sci 2000 9 1 59 78 10.1088/0963-6625/9/1/304 11624282

[B15] Carducci A Alfani S Sassi M Cinini A Calamusa A Mass media health information: quantitative and qualitative analysis of daily press coverage and its relation with public perceptions Patient Educ Couns 2011 82 475 478 10.1016/j.pec.2010.12.025 21288683

[B16] Prue CE Lackey C Swenarski L Gantt JM Communication monitoring: shaping CDC’s emergency risk communication efforts J Health Commun 2003 8 sup1 35 49 10.1080/713851975 14692571

[B17] Rutten LJF Arora NK Bakos AD Aziz N Rowland J Information needs and sources of information among cancer patients: a systematic review of research (1980-2003) Patient Educ Couns 2005 57 3 250 261 10.1016/j.pec.2004.06.006 15893206

[B18] McMullan M Patients using the internet to obtain health information: how this affects the patient–health professional relationship Patient Educ Couns 2006 63 1 24 28 16406474 10.1016/j.pec.2005.10.006

[B19] Rice RE Influences, usage, and outcomes of internet health information searching: multivariate results from the Pew surveys Int J Med Inf 2006 75 1 8 28 10.1016/j.ijmedinf.2005.07.032 16125453

[B20] Fox S Jones S The social life of health information 2009 Washington, DC: Pew Internet & American Life Project

[B21] Chung JE Patient–provider discussion of online health information: results from the 2007 Health Information National Trends Survey (HINTS) J Health Commun 2013 18 6 627 648 10.1080/10810730.2012.743628 23590202

[B22] Eysenbach G Powell J Kuss O Sa E Empirical studies assessing the quality of health information for consumers on the world wide web: a systematic review JAMA 2002 287 20 2691 2700 10.1001/jama.287.20.2691 12020305

[B23] Willis BC Wortley P Nurses’ attitudes and beliefs about influenza and the influenza vaccine: a summary of focus groups in Alabama and Michigan Am J Infect Control 2007 35 1 20 24 10.1016/j.ajic.2006.07.009 17276787

[B24] Hilton S Bedford H Calnan M Hunt K Competency, confidence and conflicting evidence: key issues affecting health visitors’ use of research evidence in practice BMC Nurs 2009 8 1 4 10.1186/1472-6955-8-4 19379494 PMC2679743

[B25] Legard R Keegan J Ward K Ritchie JE, Lewis J In-depth interviews Qualitative research practice 2003 London: Sage 138 169

[B26] Carr ECJ Worth A The use of the telephone interview for research Nurs Times Res 2001 6 1 511 524

[B27] Bloor M Frankland J Thomas M Robson K Focus groups in social research 2001 London: Sage Publications

[B28] Lincoln YS Guba E Naturalistic inquiry 1985 Beverly Hills: Sage Publications

[B29] Breuer F Roth W-M Subjectivity and reflexivity in the social sciences: epistemic windows and methodical consequences Forum Qualitative Sozialforschung/Forum: Qualitative Social Res 2003 4 2 25

[B30] Verbeke W Viaene J Guiot O Health communication and consumer behavior on meat in belgium: from BSE until dioxin J Health Commun 1999 4 4 345 357 10.1080/108107399126869 10790789

[B31] Sinaceur M Heath C Cole S Emotional and deliberative reactions to a public crisis: mad cow disease in france Psychol Sci 2005 16 3 247 254 10.1111/j.0956-7976.2005.00811.x 15733207

[B32] Department of Health Creating a patient-led NHS: delivering the NHS improvement plan 2005 London: TSO

[B33] Department of Health Equity and excellence: liberating the NHS 2010 London: TSO

[B34] Lee C-J Does the internet displace health professionals? J Health Commun 2008 13 5 450 464 10.1080/10810730802198839 18661387

[B35] Ahmad F Hudak PL Bercovitz K Hollenberg E Levinson W Are physicians ready for patients with Internet-based health information? J Med Internet Res 2006 8 3 e22 10.2196/jmir.8.3.e22 17032638 PMC2018833

[B36] Mayer G Villaire M Health literacy: an opportunity for nurses to lead by example Nurs Outlook 2011 59 2 59 60 10.1016/j.outlook.2011.01.006 21402199

[B37] Ferguson LA Pawlak R Health literacy: the road to improved health outcomes J Nurse Pract 2011 7 2 123 129 10.1016/j.nurpra.2010.11.020

[B38] Nilsson Kajermo K Alinaghizadeh H Falk U Wandell P Tornkvist L Psychometric evaluation of a questionnaire and primary healthcare nurses’ attitudes towards research and use of research findings Scand J Caring Sci 2013 doi: 10.1111/scs.12037 10.1111/scs.12037 23517064

[B39] Borglin G Fagerström C Nursing students’ understanding of critical thinking and appraisal and academic writing: a descriptive, qualitative study Nurse Educ Prac 2012 12 6 356 360 10.1016/j.nepr.2012.04.009 22633117

[B40] Veeramah V Utilization of research findings by graduate nurses and midwives J Adv Nurs 2004 47 2 183 191 10.1111/j.1365-2648.2004.03077.x 15196192

[B41] Cleary M Ferguson C Jackson D Watson R Social media and the new e-professionalism Contemp Nurse 2013 45 2 152 154 24422224

[B42] Ferguson C It’s time for the nursing profession to leverage social media J Adv Nurs 2013 69 4 745 747 10.1111/jan.12036 23488814

[B43] Robinson A Coutinho A Bryden A McKee M Analysis of health stories in daily newspapers in the UK Public Health 2013 127 1 39 45 10.1016/j.puhe.2012.10.001 23219265 PMC7111686

[B44] Kahan DM Jenkins‒Smith H Braman D Cultural cognition of scientific consensus J Risk Res 2010 14 2 147 174

[B45] Kahan D Braman D Cohen G Gastil J Slovic P Who fears the HPV vaccine, who doesn’t, and why? An experimental study of the mechanisms of cultural cognition Law Hum Behav 2010 34 6 501 516 20076997 10.1007/s10979-009-9201-0

[B46] Frewer LJ Miles S Marsh R The media and genetically modified foods: evidence in support of social amplification of risk Risk Anal 2002 22 4 701 711 10.1111/0272-4332.00062 12224744

[B47] Mcinerney C Bird N Nucci M The flow of scientific knowledge from lab to the lay public: the case of genetically modified food SciCommun 2004 26 1 44 74

[B48] Kasperson RE Kasperson JX Pidgeon NF Slovic P Pidgeon NF, Kasperson RE, Slovic P The social amplification of risk: assessing fifteen years of research and theory The social amplification of risk 2003 Cambridge: Cambridge University Press

[B49] Petts J Niemeyer S Health risk communication and amplification: learning from the MMR vaccination controversy Health, Risk & Soc 2004 6 1 7 23 10.1080/13698570410001678284

[B50] Poltorak M Leach M Fairhead J MMR “choices” in Brighton: understanding public engagement with vaccination science and delivery 2004 Brighton: IDS Working Paper 224

[B51] Loewenstein GF Weber EU Hsee CK Welch N Risk as feelings Psychol Bull 2001 127 2 267 286 11316014 10.1037/0033-2909.127.2.267

[B52] Västfjäll D Slovic P Robinson MD, Watkins ER, Harmon-Jones E Cognition and emotion in judgment and decision making Handbook of cognition and emotion 2013 New York: Guilford Press 252 271

[B53] Epstein S Integration of the cognitive and the psychodynamic unconscious Am Psychol 1994 49 709 724 8092614 10.1037//0003-066x.49.8.709

[B54] Nelson WL Han PKJ Fagerlin A Stefanek M Ubel PA Rethinking the objectives of decision aids: a call for conceptual clarity Med Decis Making 2007 27 5 609 618 10.1177/0272989X07306780 17873251

[B55] Carper BA Fundamental patterns of knowing in nursing Adv Nurs Sci 1978 1 1 13 24 10.1097/00012272-197810000-00004 110216

[B56] Rycroft-Malone J Harvey G Seers K Kitson A McCormack B Titchen A An exploration of the factors that influence the implementation of evidence into practice J Clin Nurs 2004 13 8 913 924 10.1111/j.1365-2702.2004.01007.x 15533097

[B57] Paley J Cheyne H Dalgleish L Duncan EAS Niven CA Nursing’s ways of knowing and dual process theories of cognition J Adv Nurs 2007 60 6 692 701 10.1111/j.1365-2648.2007.04478.x 18039256

[B58] De Vries M Fagerlin A Witteman HO Scherer LD Combining deliberation and intuition in patient decision support Patient Educ Couns 2013 91 2 154 160 10.1016/j.pec.2012.11.016 23265430

[B59] Kahneman D Thinking fast and slow 2011 New York: Farrar, Straus and Giroux

[B60] Jeong S-H Cho H Hwang Y Media literacy interventions: a meta-analytic review J Commun 2012 62 3 454 472 10.1111/j.1460-2466.2012.01643.x 22736807 PMC3377317

[B61] Aufderheide P Media literacy: a report of the national leadership conference on media literacy 1993 Aspen, CO: Aspen Institute

[B62] Tao M Teng Y Shao H Wu P Mills EJ Knowledge, perceptions and information about hormone therapy (HT) among menopausal women: a systematic review and meta-synthesis PLoS One 2011 6 e24661 10.1371/journal.pone.0024661 21949743 PMC3174976

[B63] World Health Organisation Effective communication during public health emergencies: a WHO handbook 2005 Geneva: World Health Organisation

